# Structure of CPV17 polyhedrin determined by the improved analysis of serial femtosecond crystallographic data

**DOI:** 10.1038/ncomms7435

**Published:** 2015-03-09

**Authors:** Helen M. Ginn, Marc Messerschmidt, Xiaoyun Ji, Hanwen Zhang, Danny Axford, Richard J. Gildea, Graeme Winter, Aaron S. Brewster, Johan Hattne, Armin Wagner, Jonathan M. Grimes, Gwyndaf Evans, Nicholas K. Sauter, Geoff Sutton, David I. Stuart

**Affiliations:** 1 Division of Structural Biology, The Wellcome Trust Centre for Human Genetics, University of Oxford, Roosevelt Drive, Oxford, Oxfordshire OX3 7BN, UK; 2 SLAC National Accelerator Laboratory, 2575 Sand Hill Road, Menlo Park, California 94025, USA; 3 Molecular Biophysics and Biochemistry, Yale School of Medicine, 333 Cedar Street, New Haven, Connecticut 06510, USA; 4 Diamond House, Diamond Light Source, Harwell Science & Innovation Campus, Didcot, Oxfordshire OX11 0DE, UK; 5 Physical Biosciences Division, Lawrence Berkeley National Laboratory, 1 Cyclotron Road, Berkeley, California 94720, USA

## Abstract

The X-ray free-electron laser (XFEL) allows the analysis of small weakly diffracting protein crystals, but has required very many crystals to obtain good data. Here we use an XFEL to determine the room temperature atomic structure for the smallest cytoplasmic polyhedrosis virus polyhedra yet characterized, which we failed to solve at a synchrotron. These protein microcrystals, roughly a micron across, accrue within infected cells. We use a new physical model for XFEL diffraction, which better estimates the experimental signal, delivering a high-resolution XFEL structure (1.75 Å), using fewer crystals than previously required for this resolution. The crystal lattice and protein core are conserved compared with a polyhedrin with less than 10% sequence identity. We explain how the conserved biological phenotype, the crystal lattice, is maintained in the face of extreme environmental challenge and massive evolutionary divergence. Our improved methods should open up more challenging biological samples to XFEL analysis.

## Abstract (web summary)

Serial femtosecond crystallography and the use of X-ray free-electron lasers (XFEL) promise to revolutionize structural biology. Here, the authors describe refinements that reduce the redundancy required to obtain quality XFEL data and report a 1.75-Å structure—not obtainable by synchrotron radiation—using less than 6,000 crystals.

## Graphical abstract

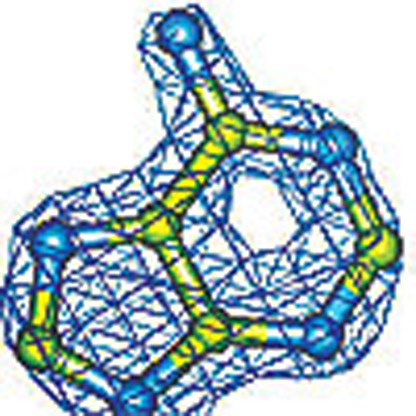

Cytoplasmic polyhedrosis viruses (CPVs) parasitize many insect species, are registered microbial insecticides^1^ and remain a problem for the silk industry^2^. CPV are protected by a robust case (polyhedron), each a tiny crystal of viral polyhedrin, whose volume varies from 10^−3^ to 10^3^ μm^3^ depending on the CPV type. Fossilized polyhedra suggest that CPV have changed little over the last 100 million years^3^, however, as expected for such ancient viral proteins, polyhedrins vary enormously in amino-acid sequence between different types of CPV. Larger polyhedra have been analysed at microfocus synchrotron beamlines^4^,^5^,^6^, but the smallest have proved refractory to such analysis, as discussed below.

Here, we measure diffraction from nanocrystals of the smallest CPV polyhedra yet characterized, using serial femtosecond crystallography (SFX) at an X-ray free-electron laser (XFEL)^7^. The very short pulse length of the XFEL beam allows us to collect useful high-resolution diffraction data at room temperature before the crystals are destroyed by the extreme peak power densities. XFEL structural determinations have been performed before, but have required very large numbers of crystals to obtain reliable high-resolution data analysis^8^,^9^,^10^,^11^. These analyses have used a Monte Carlo method whereby data from the individual crystals are simply averaged together. We apply more sophisticated methods of SFX data analysis and show that the quality of the measurements can be improved, allowing us to obtain one of the highest-resolution structures published to date (1.75 Å) from an XFEL, using 5,787 crystals of CPV type 17 (CPV17), fewer than previously required for high-resolution analysis^8^,^9^,^10^,^11^. We are able to determine the structure by molecular replacement, which in our hands fails on a lower-resolution 100 K data set of CPV17 collected at a synchrotron. However, given the XFEL analysis we are able to solve the 100 K structure and we find, unexpectedly, that the crystals remain isomorphous between 100 and 293 K, despite a ~1% alteration in the cell length, although low temperature ablates covalent disulphide bonds, which brace the room temperature lattice. The structural core of the molecule is similar to that observed for a related polyhedrin, despite less than 10% sequence identity. However, we find that, in addition, there are a number of smaller more variable non-core structural modules. The polyhedrin building blocks and their arrangement in these crystals remain similar to those observed before, but sequence variation in the structural modules allows plasticity and evolutionary robustness. Our methods provide a new physical model for XFEL diffraction data, which allows a more accurate estimation of the actual signal. The need for such improved methods is indicated by the recent papers from White^12^ and Kabsch^13^. White devise a model for XFEL diffraction data, which has similarities with the one we describe below, but only describe its application to simulated data. Kabsch defines a term ‘Ewald offset correction’, which is similar to our ‘partiality’, and applies it successfully to synchrotron still images. We here demonstrate the power of such a method tailored to and applied to real XFEL data, revealing the promise of such improved models to allow XFELs to tackle more challenging biological samples.

## Results

### XFEL data collection and analysis

CPV17 polyhedrin nanocrystals were grown in *Spodoptera frugiperda* (Sf9) cells, purified and diffraction data were collected at the Coherent X-ray Imaging (CXI) beamline, Linac Coherent Light Source (LCLS). A suspension of crystals was injected into the beam *in vacuo*^14^, presumably at close to room temperature, and femtosecond diffraction snapshots were recorded at 120 frames per second. Initial processing used cctbx.xfel^15^,^16^ to identify useful images and provide preliminary crystal orientation matrices^15^. Within 20 min 5,787 indexable images had been collected at ~1.46 Å wavelength that were used for further analysis.

Accurate modelling of the diffraction underpins the huge success of protein crystallography, using sophisticated models developed over many years^17^,^18^. However, fundamental differences in SFX diffraction experiments prevent the direct application of these methods. Most XFEL analyses have used the so-called ‘Monte Carlo’ method^19^, where potentially observable reflections are summed and the orientation matrix is modelled imprecisely, requiring the use of massive numbers of XFEL images to obtain accurate measurements because the integratable spots are not accurately predicted. We have developed a refinement method that significantly improves the crystal orientations, and a model for partiality prediction that takes account of crystal size, orientation, mosaicity and the spectral properties of the self-amplified spontaneous emission (SASE) X-ray pulse^16^,^20^. This allows a quantification of the relative fraction of the beam energy captured by each reflection (termed partiality). Here we present a first implementation of these models to the analysis of the CPV17 data. Recent papers by White^12^ and Kabsch^13^ developed related models, but did not apply those models to the analysis of experimentally obtained XFEL data.

**Figure 1:**
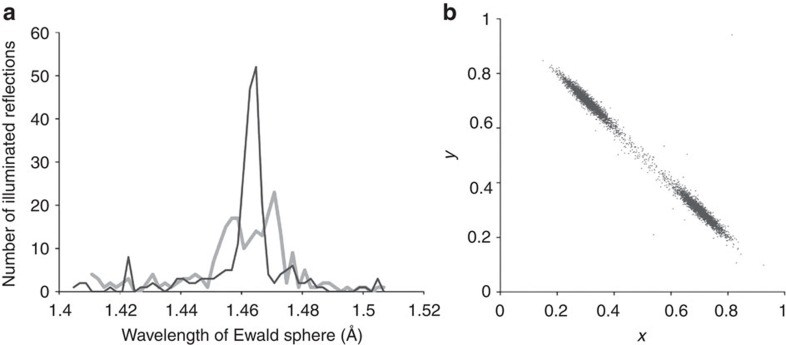
XFEL data analysis. (**a**) Distribution of Ewald sphere wavelengths for all reflections on a single image, before (grey, thick) and after (black, thin) orientation matrix refinement. Refinement resulted in a 0.117° rotational shift in the orientation model. (**b**) Cluster algorithm by a modified version of algorithm of Brehm and Diederichs^24^, showing positions of final artificially defined vectors corresponding to individual images (see Methods). Note the vectors fall into two sets, clearly separating the two indexing choices.

As an essential first step, we refined the orientation matrices of all crystals by an iterative process. Diffraction was modelled by describing the X-ray beam using an inflated energy bandwidth and infinitely small reciprocal lattice points (rlps), providing a ‘net’ to catch all possible reflections. These potential reflections were integrated, and strong reflections deemed ‘hits’. Refinement initially aimed to sharpen the histogram of frequency of hits *vs* wavelength by slightly adjusting the crystal orientation around the two axes orthogonal to the beam. This method worked well, a typical distribution before and after refinement is shown in Fig. 1a. The average combined angular correction was 0.075°. This refinement also optimized the estimate of the mean wavelength for each pulse. In contrast, the Kabsch paper reorients the crystal to minimize the rlps' distance from a nominally monochromatic Ewald sphere. An experimental model was also invoked, accounting for spot size (derived from crystal size), X-ray bandwidth and crystal mosaicity, each refined to best predict the observed selection of hits. For integration, spot positions were centred on the local maximal peak to allow for imperfect metrology. Images were metrology-corrected by cctbx.xfel^16^ and integrated using DIALS^21^. The bandwidth of the pulse was derived from the hit count histogram (Methods). The spot diameter refined to 1.8 × 10^−4^ Å^−1^, corresponding to a crystal size of ~0.5 μm, plausible based on electron microscopy analysis of the crystals. A mosaicity of 0.03° was used, which is consistent with synchrotron data but may also be an overestimate. Spots were generally well predicted (Supplementary Fig. 1). The limiting resolution for each image was chosen as the highest-resolution shell with *I*/*σ*(*I*) of 2.0, which almost always integrated to the edges of the image.

Partiality was calculated for each reflection. This corresponds to the fraction of the total energy of the X-ray pulse contributing to the diffraction compared with the maximum that could have been used (see Methods). This model differs from that used for the rotation method^22^, does not require further Lorentz correction and a polarization correction was not applied. For all further statistical analysis and calculations, reflection intensities and weights were divided by their partiality, and ~113 reflections per image with partiality of 0.3 or below were discarded since they were poorly estimated (the large number of reflections discarded reflects the previous overprediction of reflections), leaving a total of 1,093,434 reflections. Statistics are shown in Table 1. The effectiveness of partiality modelling is shown in Supplementary Fig. 2. Without any partiality modelling, the CC_1/2_ parameter^23^ is 97.2% across all resolution shells. Using a partiality cutoff of 0.3—but not dividing by partiality—increases CC_1/2_ to 97.4%, while dividing accepted reflections by their partiality increases it to 98.3%.

The crystals belong to space group I23 and are therefore indexed ambiguously—the indexing schemes (h, k, l) and (k, h, −l) are geometrically indistinguishable. We used the method of Brehm and Diederichs^24^ to break this ambiguity while including the correlation between the alternative indexing choice, thereby doubling the number of pairwise comparisons (see Methods). Figure 1b shows the clear separation of crystals into the two indexing sets that were transformed to a consistent indexing set and simply merged with crude scale factors applied to give an interim reference data set. Scale factor refinement for each image was then made against this interim reference data set and the weakest images discarded before merging. The CC_1/2_ dropped below 0.3 at a resolution of 1.75 Å, taken as the nominal resolution (Supplementary Fig. 3). The *R*_split_ value was calculated between the two indexing halves of the data set. Over this resolution range the average *R*_split_ was 11.7% using 5,554 images. The effect of set size is shown in Supplementary Fig. 3.

### Synchrotron cryo-crystallography

In parallel to the XFEL data collection, crystals were analysed at 100 K on a microfocus synchrotron beamline (I24, Diamond Light Source). Data from 768 crystals yielded a moderate resolution data set with a CC_1/2_ of 0.3 at 2.15 Å resolution (Supplementary Fig. 3a)^25^. To accurately determine changes in unit cell dimensions between room temperature and 100 K, powder patterns were collected at Diamond Light Source, which showed a contraction of the unit cell dimension on cooling from 106.1 to 104.9 Å (values used as the cell dimensions for the XFEL and synchrotron analyses, respectively). We consider the unit cells to be highly uniform at each temperature since the powder rings are tightly defined to the highest recorded resolution. Despite this temperature-dependent variation in the crystal lattice, the correlation in intensities between the XFEL and synchrotron data was excellent (90.9% overall, Supplementary Fig. 3a). This surprising isomorphism is discussed below. In the absence of a good molecular replacement model, these synchrotron-source data were not, in our hands, sufficient to support structure determination.

### Structure determination from XFEL data

**Figure 2:**
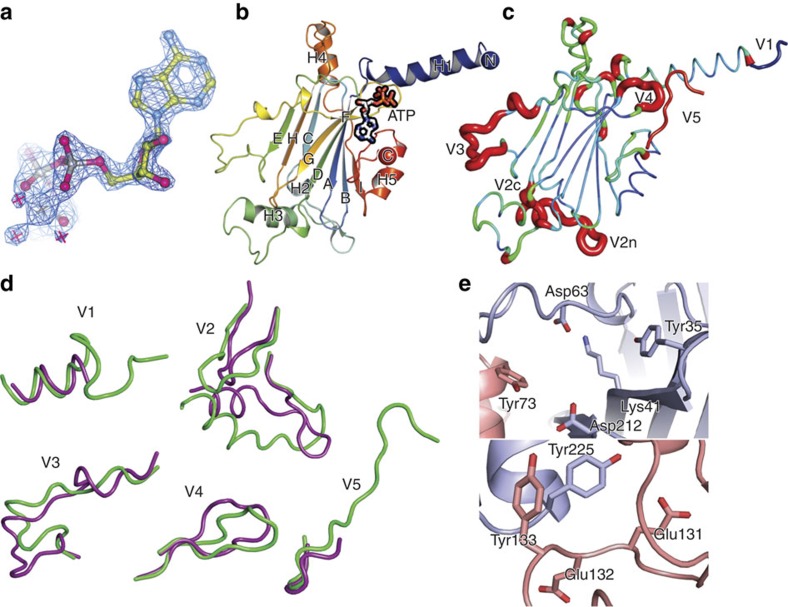
Structure of CPV17 polyhedrin. (**a**) Electron density around the ATP moiety contoured at 1.3 *σ*. (**b**) Cartoon of the CPV17 polyhedrin subunit coloured from blue to red, N terminus to C terminus that are denoted by spheres. Secondary structure elements are labelled and the ATP molecule shown as sticks. (**c**) Comparison of the CPV17 and CPV1 polyhedrin structures. The molecules were aligned with program SHP^43^. Both colour and tube thickness represent r.m.s. distance (r.m.s.d.) of equivalent C-alpha atoms (thin, blue: r.m.s.d.<1.0 Å, green, thicker: 1.0–2.5 Å, orange, thickest: >2.5 Å). Unaligned regions are coloured red and displayed with exaggerated thickness. Variable regions are labelled v1–v5 with the N- and C-terminal extensions of CPV1 drawn. (**d**) Comparison of variable regions between CPV17 (magenta) and CPV1 (cyan). (**e**) The interface between monomers (coloured differently) in the crystal is rich in tyrosines.

The structure of CPV17 was solved by molecular replacement using the higher-resolution XFEL data. Manual rebuilding and refinement rapidly reduced R_work_/R_free_ from 56.5%/54.1% to 12.2%/15.4% (Table 1). Beyond 2.0 Å resolution, at which point *R*_split_ exceeds 20%, the *R*_work_ and *R*_free_ increase significantly. However, the quality of the electron density meets that expected for a high-quality 1.75-Å resolution analysis (Supplementary Movie 1), for instance allowing the unambiguous identification of an unexpected bound adenosine triphosphate (ATP) (Fig. 2a). Using this room temperature structure the synchrotron 100 K structure was solved trivially and refined to 2.2 Å (Table 2).

### CPV17 polyhedrin shows similarity to CPV1 polyhedrin

**Figure 3:**
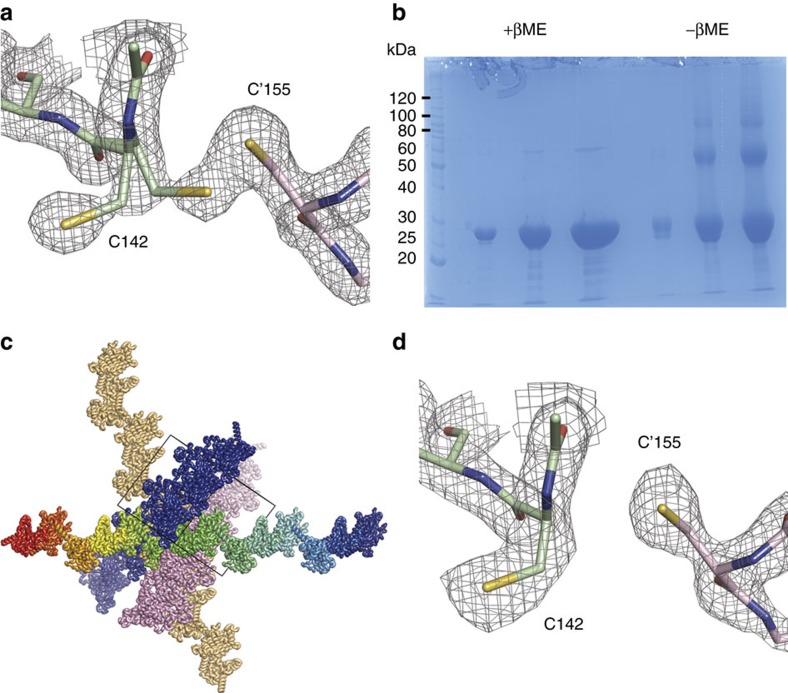
Disulphide bonds link chains of CPV17 polyhedrin molecules. (**a**) Electron density around C142 (pale green) and C155 (pink) from a related molecule in the crystal contoured at 1.0 *σ* for CPV17 at 293 K. (**b**) SDS–polyacrylamide gel electrophoresis analysis of CPV17 crystals with and without reducing agent 2-mercapto-ethanol. (**c**) Visualization of the intermolecular disulphide-linked helical strings going through the crystal lattice of CPV17. Four helical strings are drawn, one with individual polyhedrin molecules coloured separately, the others coloured pink, cream and blue. (**d**) Electron density around C142 (pale green) and C155 (pink) from a related molecule in the crystal contoured at 1.0 *σ* for CPV17 at 100 K.

Two hundred and thirty-six of the 237 CPV17 polyhedrin residues were well defined in the XFEL electron density map; only the N-terminal residue could not be seen (Fig. 2b). In addition, an ATP molecule with bound Mg^2+^ was identified (Fig. 2a). Overall, the protein fold is remarkably similar to that of CPV1 polyhedrin with the two protein crystals sharing the same I23 space group^26^. 191 Cα atoms can be superposed with root mean square (r.m.s.) deviation 1.4 Å (Fig. 2c,d), despite less than 10% residues being identical in the sequence (Supplementary Fig. 4). In addition, the molecules are placed almost identically in the crystal lattice (0.6 Å displacement between the cores of the molecules). This is reflected in the similarity in the unit cell dimensions (*a*=104.9 and 102.8 Å for CPV17 and CPV1, respectively). There are five small structural modules that show significant structural variation between the two molecules (V1–5, Fig. 2c,d). V1 is the N-terminal region, which in CPV1 is extended. V2 comprises some 20 residues forming a ‘cap’ at one end of the molecule, containing two portions of polypeptide (V2n and V2c, in Fig. 2c). V3 forms a protrusion on the flank of the molecule; V4 interacts with a bound ATP in both CPV1 and CPV17 (described in detail below), and V5 is the C-terminal region, also extended in CPV1. In the alkali conditions of the insect mid-gut (pH 10.5 or greater), the crystal dissolves to release the infectious virus^27^. It has been postulated that the pH susceptibility arises from the deprotonation of tyrosine residues^4^. CPV17 harbours several tyrosines in environments where they could contribute to pH-mediated crystal disassembly (Fig. 2e), and are partly conserved in CPV1 (ref. 6). An unexpected feature of the CPV17 crystal is an incompletely (50%) formed intermolecular disulphide bond (Fig. 3a,b), which assembles helical strings of covalently linked molecules, presumably contributing to the mechanical stability of the crystal (Fig. 3c). Splitting of disulphide bonds is accelerated in the high pH carbonate environment of the insect mid-gut^28^, facilitating dissolution. Interestingly, this disulphide is not observed in the 100-K structure (Fig. 3d). To test whether this was due to radiation damage, we assembled two data sets, one derived from the first half of the images collected from each crystal and the other derived from the second half of the data from each crystal. The two electron density maps were essentially identical, indicating that in difference was not due to radiation damage. The B-factors of sulphur atoms in the room temperature structure suggest that XFEL data collection also inflicts little radiation damage. A possible explanation for this temperature-dependent difference in structure is presented below.

### Nucleoside triphosphates stabilize the crystal lattice

**Figure 4:**
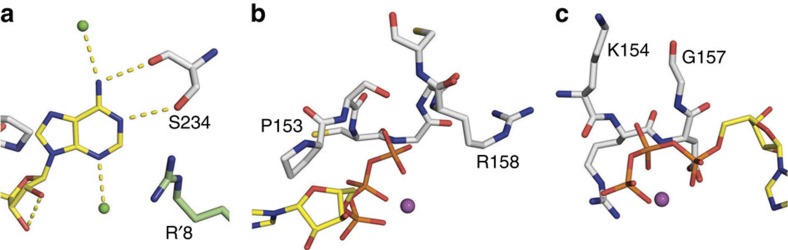
ATP interactions. (**a**) Interactions of the base moiety of ATP of CPV17. Arg 8 (coloured green) is from a related molecule. Alignment of the ATP molecule for (**b**) CPV17 and (**c**) CPV1.

The polyhedrins of CPV1 and CPV17 possesses a conserved structural core with modules of variable structure associated with it. One of these modules (V4) is involved in ATP association in different ways in CPV1 and CPV17. Crystals of CPV1 polyhedrin harbour various nucleoside triphosphates at monomer interfaces^4^. Unexpectedly CPV17 crystals contain only a single ATP molecule, positioned in a broadly similar position to an ATP found in CPV1. The ATP is well ordered in CPV17, with specific adenine–amine interactions ensuring ATP specificity (GTP is excluded by steric hindrance) (Fig. 4a), however, the triphosphate-Mg^2+^ moiety is the most tightly located, making a set of interactions formed primarily by a ring of backbone amide nitrogen atoms flanked by an arginine side chain (Fig. 4b). This configuration is similar to that locating the corresponding ATP in CPV1 polyhedra (Fig. 4c), although the phosphates are translated by some 6 Å, due to a shift in the position of the V4 loop, and the ATP molecule is rotated by ~180°, so that the mechanism of nucleotide selection is quite different. However, despite the variation in such modules (which in CPV17 compensate for the absence of other nucleoside triphosphates found in CPV1), the fundamental assembly of the I23 lattice remains unchanged.

### Temperature changes induce lattice swelling/shrinking

Comparison of the structures at room temperature and 100 K demonstrates the response of the crystals to extreme environmental changes. The structures are very similar (r.m.s.d. in Cα 0.26 Å after rigid body superposition). Nevertheless, there is a ~1% reduction in the cell dimensions on cooling. Previous studies^29^ indicated that protein crystals accommodate such lattice changes by a number of switches in the intermolecular interactions within the crystal. To see whether this occurs in the CPV17 polyhedra, we artificially increased the unit cell for the 100 K data to match that of the XFEL data (thereby isotropically ‘swelling’ the structure of the monomer) and performed simple restrained refinement of the resulting structure. Remarkably, these structures were then almost indistinguishable (r.m.s.d. in Cα 0.17 Å). This suggests that the complete crystal has evolved as a robust material able to simply expand or contract slightly in a surprisingly homogenous way in the face of environmental insult, without repacking. This accords with the lattice being a key biological phenotype. Nevertheless, the strain in the (unphysiological) 100-K lattice is enough to sever the partially formed disulphide bond.

## Discussion

In summary, orientation matrix refinement, careful selection of illuminated spots, partiality modelling and image scaling have markedly reduced the redundancy required to obtain a high-quality XFEL data set at high resolution, allowing perhaps the most challenging XFEL structure determination reported to date. How general are these results? While our nanocrystals possess high symmetry (point group 23) providing a high multiplicity of unique reflections, they also possess low mosaicity (~0.03**°**) reducing the number of reflections per image compared with that which might be expected for many protein crystals. We therefore believe that by adopting procedures such as those presented here, the number of XFEL images required for structure determination will be markedly reduced compared with that required previously^8^,^9^. This, together with improved sample preparation and presentation techniques^30^, may render the method attractive for challenging problems, and should greatly facilitate experimental phasing^19^. In addition, less beam time will be required to collect sufficient data, in this case only 20 min. Finally, despite attenuating the beam so that only 2% of the possible X-ray photons were used, the 1.75 Å electron density map was of exceptional quality, suggesting that SFX has unexploited potential for high-resolution analyses.

## Methods

### Sample preparation

The polyhedrin gene for *Uranotaenia sapphirina* CPV17 was synthesized by GeneArt (Life Technologies) based on the Gene Bank sequence AY876384 (ref. 31). The polyhedrin gene was amplified and inserted into the transfer vector pBacPAK9 (Clontech). Recombinant baculovirus was produced by co-transfection of linearized baculovirus DNA and the transfer vector following a standard protocol^32^. Expression and purification of polyhedra followed the protocol described previously^26^. Cells were lysed, polyhedra pelleted at 500*g* and resuspended in water for storage.

### Serial femtosecond crystallography

SFX experiments were carried out at the Linac Coherent Light Source of the SLAC National Accelerator Laboratory (Menlo Park, CA, USA), on the CXI instrument^33^. X-ray pulses of 50 fs duration containing ~2.0 × 10^12^ photons per pulse at a wavelength of 1.46 Å (this mean pulse wavelength was initially derived from the tuned energy of the laser) were focused by Kirkpatrick–Baez mirrors to 2–3 μm diameter at the interaction point. The sample–detector distance was 90.9 mm. The photon beam parameters are summarized in Table 1. The crystal slurry was diluted to provide ~3 × 10^9^ crystals per ml to provide a hit rate of ~4% (ref. 15)). The crystals were injected into the XFEL beam in water using a Gas Dynamic Virtual Nozzle^14^ focused to a diameter of a few μm at a flow rate of 30 μl min^−1^. During measurement a rotating syringe device^34^ was used to avoid crystal settling. The injector was positioned to intersect the X-ray beam, before the Rayleigh break-up of the jet into drops. Single-shot diffraction patterns were recorded at 120 Hz while the liquid jet was flowing. The X-ray beam incident on the jet was attenuated to 2% of its original intensity. Data were recorded on a Cornell-Stanford pixel array detector^10^.

### Refinement of orientation matrix

A total of 144,803 images from ~20 min of data collection (which used 632 μl of crystal suspension) were analysed with cctbx.xfel^16^ to determine initial orientation matrices. A total of 5,787 images could be indexed with the software available at that time. To ‘catch’ a complete set of observed reflections, the image was integrated with an inflated bandwidth (*λ*±3.5%), and those predicted reflections whose intensity exceeded 150 ADU (analogue-to-digital units) after integration were selected. At this stage rlps were modelled as delta functions. Orientation matrices were refined to minimize the spread of wavelengths required to satisfy the Bragg condition for this observed set. Orientation matrices were rotated about two axes orthogonal to the beam, each initially rotated by 0.5°, then halving the interval step upon finding local minima. For each orientation a histogram was calculated of the number of reflections versus the wavelength at which they were excited. The target function initially maximized reflection count and minimized distribution spread, where the parameters are altered to result in a minimization of the value of the target function. After the interval step had been halved three times, the target function was changed to be the quality of fit to a Gaussian model of wavelength distribution. Refinement was terminated when the rotational step size reached 0.001°.

### Partiality model

Like the recent paper from White^12^, we treat partiality as arising from the intersection of a rlp with a finite volume with a set of Ewald spheres of different radii, representing the range of incident X-ray wavelengths. Our partiality model rests on defining a partiality of 1 as a reflection that maximally excites all wavelengths of the X-ray pulse. In this context, the extent of excitation is defined as the cross-sectional area of the rlp that intersects the Ewald sphere, relative to the area of a central section of that rlp. Thus, notionally, the 0,0,0 rlp will have a partiality of essentially 1, since the Ewald spheres are almost flat and pass centrally through the rlp. In practice we split this into two components, we first model the portion of the reflection that lies between the limiting spheres, and then integrate across the wavelengths that the rlp excites, using a Gaussian model for the wavelength distribution of the photons in each pulse. To deconvolute the width of this wavelength distribution from the Gaussian distribution we have fitted empirically for each pulse (which includes the combined effect of bandwidth, crystal mosaicity and finite crystal size), we multiply the s.d. by 0.27, found to be effective after a few manual trials. To model partiality, the Gaussian distribution was set to 0 beyond two s.d. from the mean, thereby defining the limiting Ewald spheres. Rlps were modelled as spherical top-hats with radii derived from crystal size and isotropic mosaicity; the intersection of these with the limiting Ewald spheres was calculated and used to derive appropriately scaled cylindrical approximations (where the cylinder base is in the plane of the tangent to the Ewald sphere, Supplementary Fig. 5). The partiality is then obtained by integrating across the wavelength distribution. The precise unreduced expression for partiality is shown in equation (1),


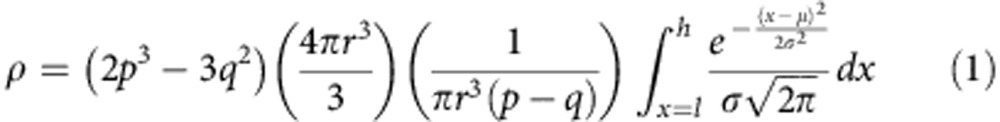


where *p* and *q* are the fractions along the diameter of a spot at which the limiting radii of the Ewald spheres intersect, *r* represents the radius of the spot after correction for mosaicity, *μ* is the mean wavelength of the beam, *σ* is the s.d. of the beam wavelength and *l* and *h* are the lower and higher wavelengths corresponding to Ewald radii two s.d. from the mean, respectively. Reflection size was calculated according to a fixed term, spot size (equal to 1.8 × 10^−5^ Å^−1^, determined by trial and error to minimize overprediction of spots), plus a mosaicity term (0.03°, as previously defined^22^), which allows variation in spot size with resolution.

The error model for the reflections combined counting statistics from integration divided by both the partiality and the scale factor for each image. Since the gain of the detector was not properly accounted for, these are not reliable.

### Integration of each image

Spot positions were computed using the refined orientation matrices and metrology^16^. Any residual errors in metrology were corrected for by centring on the highest count pixel in a 5 × 5-box around this point. Images were integrated using simple two-dimensional (2D) integration using the DIALS software package. This 2D ordinate analysis method^35^ will slightly overestimate weak data. The resolution of the image was taken as the highest-resolution shell where *I*/*σ*(*I*) reached 2.0. For each image the raw integration count was recorded along with the partiality estimated for that given reflection.

### Separation of indexing hands by cluster analysis

Each image was assigned an individual 2D vector, 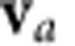, where *a* is the image number. Both components of the vector are randomly assigned between 0 and 1 according to the cluster algorithm previously defined^24^. The algorithm has been modified to include a second term, corresponding to the inverted indexing hand. *r*_*ab*_ is the correlation coefficient between image numbers *a* and *b*, (equation 3), while the correlation between the (*h*, *k*, *l*) reflection of one image with the (*k*, *h*, *l*) reflection of the other is represented by 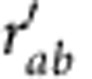. These vectors were minimized using an L-BFGS algorithm^36^ (equation (2)).


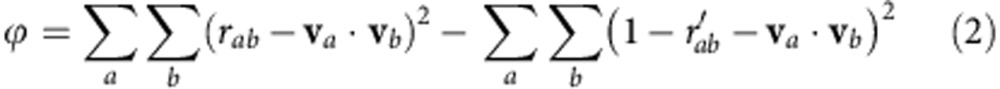


Correlation coefficients *r*_*ab*_ and 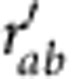 lie between 0 and 1, and the formula is modified to ensure that the regression line passes through the origin (equation (3)). This allows a meaningful correlation coefficient to be generated from just two shared reflections. If the value of *r*_*ab*_ or 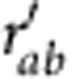 is imaginary, then it is reassigned a value of 0.


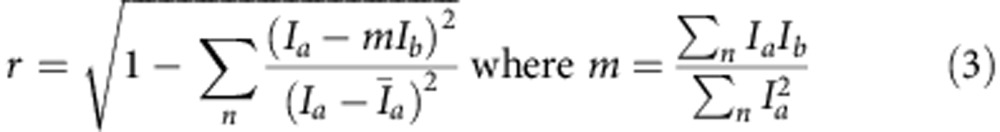


Correlation coefficients are generated from intensities that have been corrected for partiality. Reflections with a partiality less than 0.3 were rejected, the remaining reflection intensities and sigma values were divided by their partiality. Beyond 1.8 Å resolution the reflection count rapidly dropped due to the smaller angle of observation on the detector.

### Scaling images

Scale factors for individual images were generated initially by adjusting the mean intensity of reflections (to 3.5 Å resolution) in each image to equal values. After rejecting 233 (3.6%) of the weakest images (those for which the mean intensity was <333 ADU) and merging, scale factors were then adjusted by calculating the gradient between each image and the initial merged data set, after remerging the data with the new scale factors the process was iterated to convergence.

### Powder diffraction

Crystals were mounted into capillary tubes for powder diffraction experiments. Data were collected at 100 K and a second data set collected at room temperature, using otherwise identical experimental conditions (the detector was not moved, the wavelength was identical and the data were collected close together in time). Accurate unit cell dimensions were calculated from the very sharp diffraction pattern rings.

### Synchrotron data collection

Polyhedra were purified as for the XFEL sample. Crystals were cryo-protected by mixing 1:1 with ethylene glycol, equilibrated for 60 s, applied to a MicroMesh mount (Mitegen, Ithaca, USA) and allowed to settle for 30 s before excess liquid was wicked away. The crystals were flash-cooled in a stream of nitrogen gas at 100 K. The X-ray beam at beamline I24, Diamond Light Source was trimmed to ~4 × 4 μm at the sample using apertures close to the crystal (D.A. and G.E., unpublished). Only 1° of data could be collected per crystal (20 images of 0.05°), due to the long exposure times required. Data collection and processing, summarized in Table 2, have been described previously^25^. The final data set comprised measurements from 768 crystals. Initial attempts at molecular replacement using these data were unsuccessful. The structure was solved by choosing the same indexing choice as the XFEL data and then positioning the XFEL model (see below) by rigid body refinement, followed by standard refinement with REFMAC5 (ref. 37).

### Molecular replacement and model refinement

Structures of seven homologues with 9–18% sequence identity with CPV17 (unpublished data and refs 6, 26) were superposed, the occupancy of each atom reduced to 1/7 and the coordinates concatenated. This agglomerated coordinate set was used as a single search model in Phaser^38^, leading to preliminary phases, and a starting map that clearly showed novel information (Supplementary Fig. 6). The model with closest sequence similarity was set to polyalanine and manually rebuilt in Coot^39^. After initial phase improvement, Buccaneer^40^ was used for partial rebuilding of the remaining model. The remainder of the refinement was completed manually, using REFMAC5 (ref. 37) and Phenix^41^. Interestingly, Phaser found a similar molecular replacement solution using the synchrotron data, but we were unable to produce a reliable refined model from that starting point, presumably because, without higher-resolution data, in the absence of non-crystallographic symmetry and with very little bulk solvent it was not possible to escape bias from the starting models. The quality of the final electron density maps is shown in Supplementary Fig. 7.

## Author contributions

G.S. prepared samples. All authors performed research. H.M.G. wrote the original software. H.M.G., G.S., N.K.S., G.E. and D.I.S. wrote the manuscript, in discussion with the other authors.

## Additional information

**Accession codes:** The coordinates and structure factors for the XFEL and synchrotron structures of CPV17 have been deposited with the RCSB under accession codes 4S1L and 4S1K, respectively, and raw data at CXIDB.org^42^.

**How to cite this article:** Ginn, H. M. *et al.* Structure of CPV17 polyhedrin determined by the improved analysis of serial femtosecond crystallographic data. *Nat. Commun.* 6:6435 doi: 10.1038/ncomms7435 (2015).

## Supplementary Material

Supplementary Information Supplementary Figures 1-7 and Supplementary References

Supplementary Movie 1 Example of final model and electron density.

## Figures and Tables

**Table 1:** XFEL data collection and refinement statistics.

	**CPV17**
Data collection	
Space group	I23
Cell dimensions	
*a*, *b*, *c* (Å)	106.1, 106.1, 106.1
*α*, *β*, *γ* (°)	90, 90, 90
Resolution (Å)	28.30–1.75(1.79–1.75)*
*R*_pim_ (%)	7.4 (33.8)
*R*_split_ (%)	11.8 (58.4)
CC_½_	98.0 (38.6)
Completeness (%)	100 (100)
Redundancy	52.4 (11.1)
	
Refinement	
Resolution (Å)	28.36–1.75
No. of reflections	20,122
*R*_work_/*R*_free_	12.2/15.4
No. of atoms	
Protein	1,914
Ligand/ion	32
Water	174
B-factors	
Protein	23.7
Ligand/ion	34.4
Water	32.7
R.m.s. deviations	
Bond lengths (Å)	0.010
Bond angles (°)	1.39

CPV17, CPV type 17; XFEL, X-ray free-electron laser.

Number of crystals used: 5,554.

^*^Highest-resolution shell is shown in parenthesis.

**Table 2:** Synchrotron data collection and refinement statistics.

	**CPV17**
Data collection	
Space group	I23
Cell dimensions	
*a*, *b*, *c* (Å)	104.9, 104.9, 104.9
*α*, *β*, *γ* (°)	90.0, 90.0, 90.0
Resolution (Å)	74.16–2.20 (2.26–2.20)*
*R*_merge_ (%)	66.5 (325.9)
*I*/σ*I*	6.4 (1.4)
Completeness (%)	99.9 (100.0)
Multiplicity	47.8 (25.4)
	
Refinement	
Resolution (Å)	74.16–2.20
No. of reflections	9,376
*R*_work_/*R*_free_	14.7%/19.9%
No. of atoms	
Protein	1,907
Ligand/ion	32
Water	146
B-factors	
Protein	22.5
Ligand/ion	50.0
Water	32.2
R.m.s deviations	
Bond lengths (Å)	0.013
Bond angles (°)	1.675

CPV17, CPV type 17.

Number of crystals used: 768.

^*^Highest-resolution shell is shown in parenthesis.
