# Supplementary material for: Structure of CPV17 polyhedrin determined by the improved analysis of serial femtosecond crystallographic data
Source: Nat Commun. 2015 Mar 9;6:6435. doi: 10.1038/ncomms7435 (PMC4403592; doi:10.1038/ncomms7435)
Supplement: Supplementary Information — Supplementary Figures 1-7 and Supplementary References [file ncomms7435-s1.pdf]

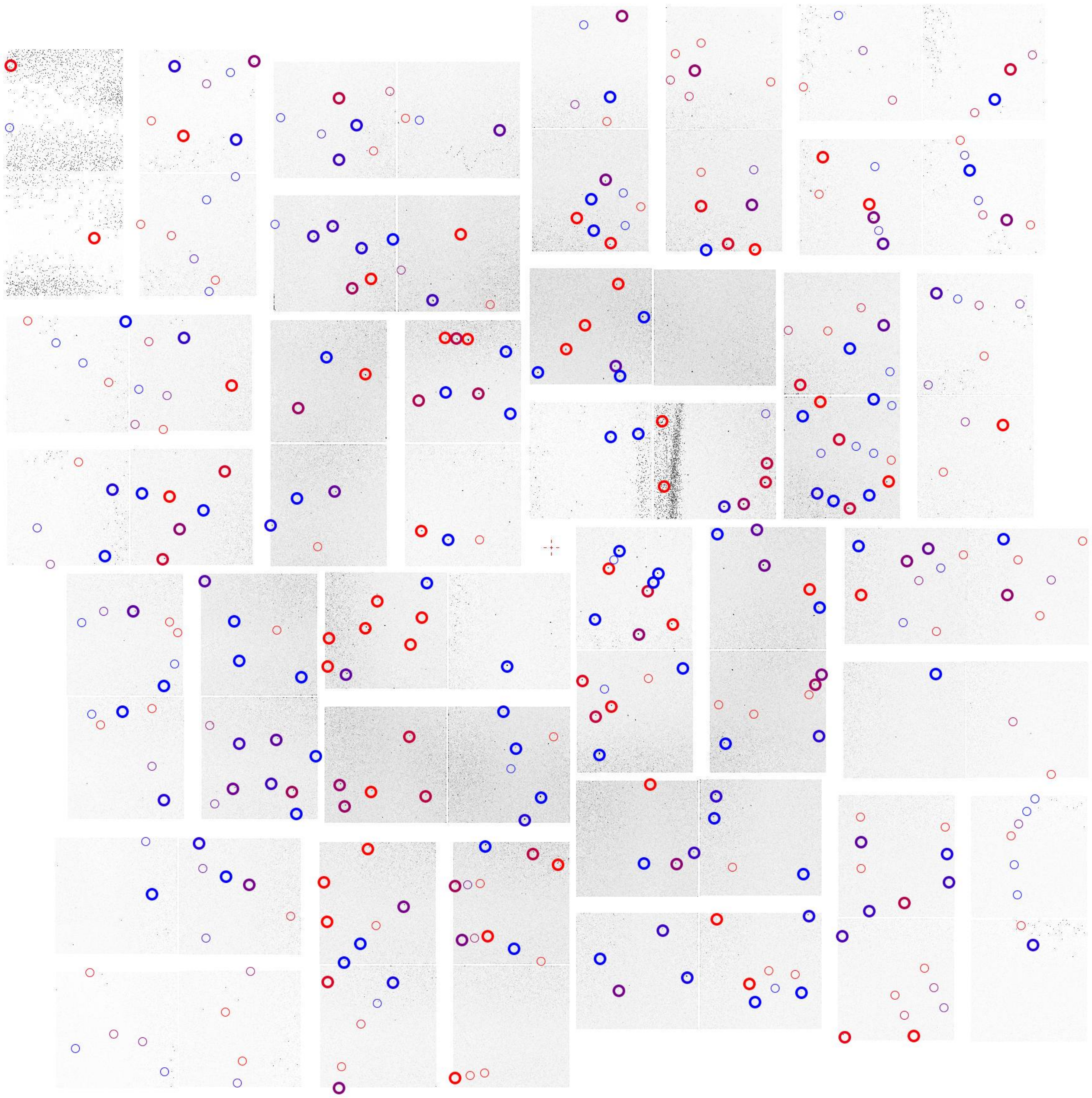

**Supplementary figure 1. Reflections integrated on a given image.** Thick lined circles represent reflections which, when integrated, have an I/σ(I) greater than 2.0. Colour of circle varies from red to blue where red represents lower Ewald sphere wavelength and blue represents higher Ewald sphere wavelengths. Note that low resolution spots have Ewald sphere wavelengths deviating significantly from the average of 1.459 Å compared to those at higher resolution.

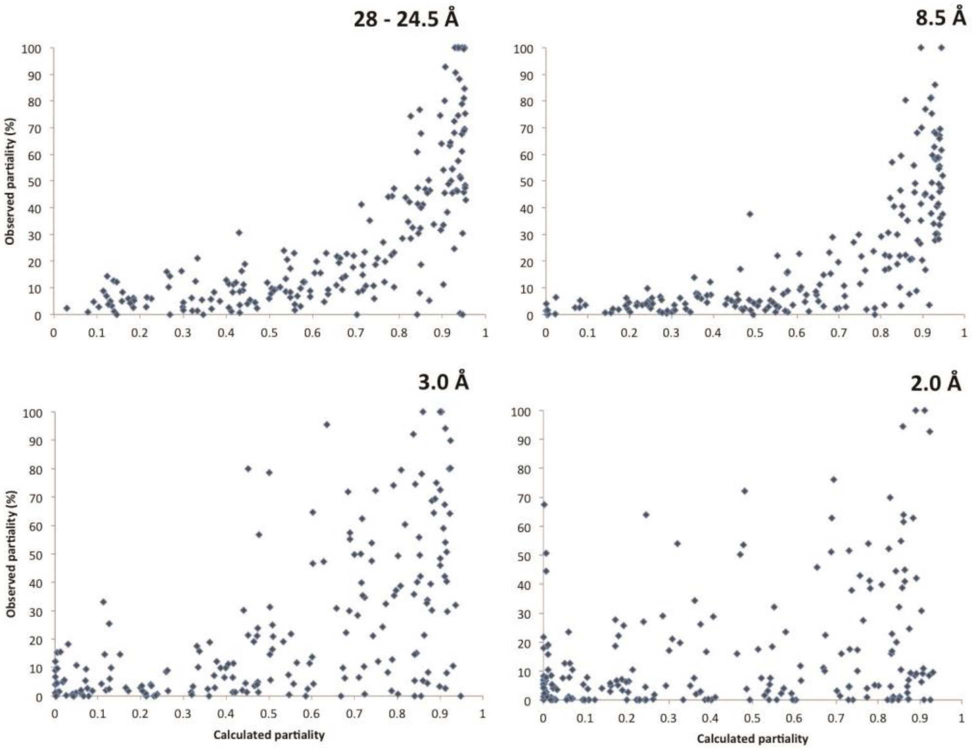


**Supplementary figure 2. Partiality analysis.** Calculated partialities *vs* percentage of maximum reflection intensity recorded for 200 reflections in each resolution bin, for reflections at resolutions 28 – 24.5 Å, 8.5 Å, 3.0 Å and 2.0 Å. Relationship between calculated and observed partiality is strongest at low resolution. Partiality model at high resolution will be hindered by residual errors in the orientation matrix. All reflections below a partiality of 0.3 are discarded during calculations and in final data set.

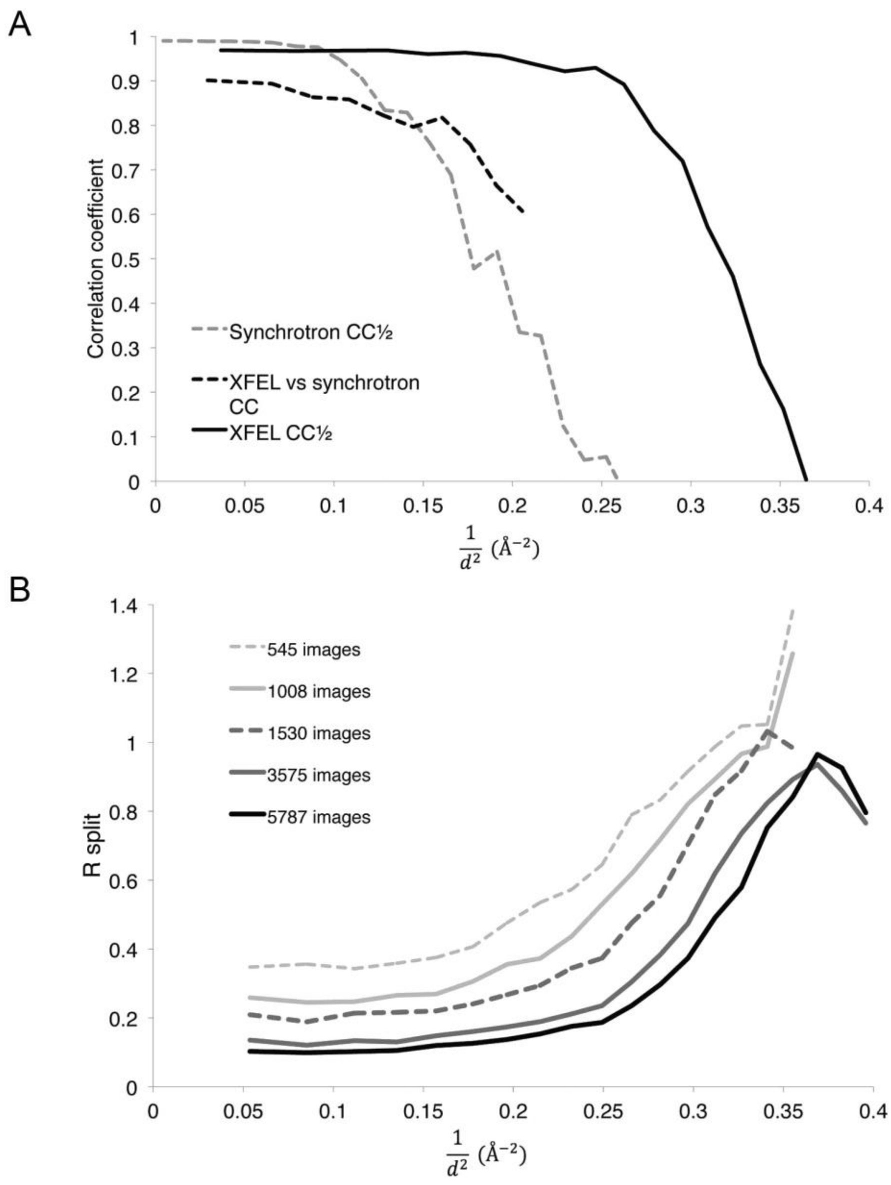


**Supplementary figure 3. Data quality.** (**a**), the black dashed line shows correlation between XFEL data set and synchrotron data set to a resolution of 2.2 Å; black line plots CC½ between the two indexing hands of the XFEL data set to the maximum recorded reflection of 1.60 Å. CC½ drops below 0.3 at 1.75 Å which is taken as the nominal resolution; grey dashed line shows CC½ for the synchrotron data, falling to 0.3 at 2.15 Å. (**b**)**,** R split *vs* resolution for increasing numbers of images.

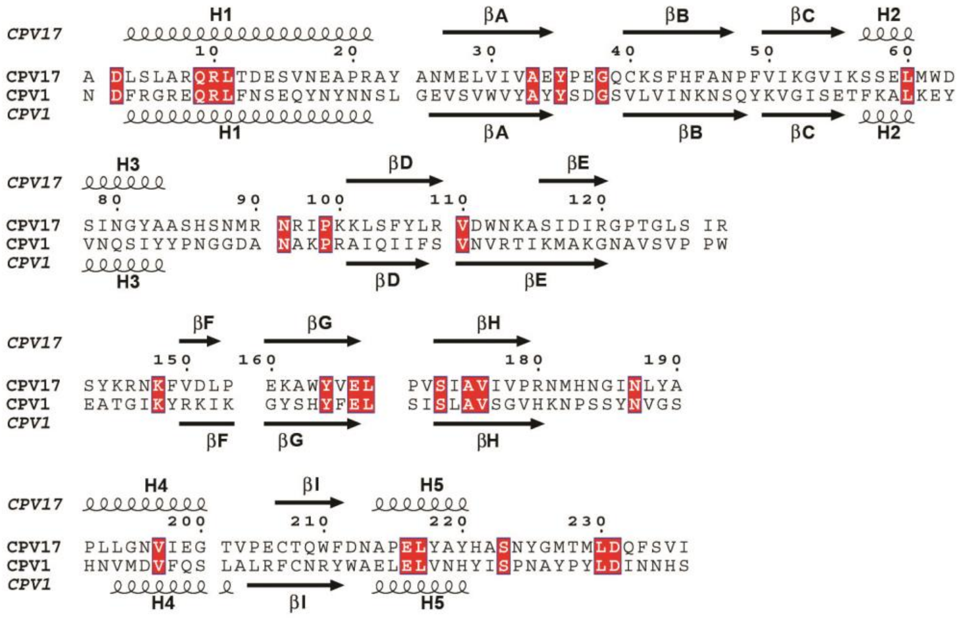


**Supplementary figure 4. Sequence alignment based on 3-D structure superposition.** CPV17 and CPV1 polyhedrin structures were superposed using SHP[1]. Equivalent residues are shown on the figure. Secondary structures are marked above and below the sequences and are labelled as in Fig. 2b. Fully conserved residues are marked in solid red boxes. The figure was visualized with ESPript[2].

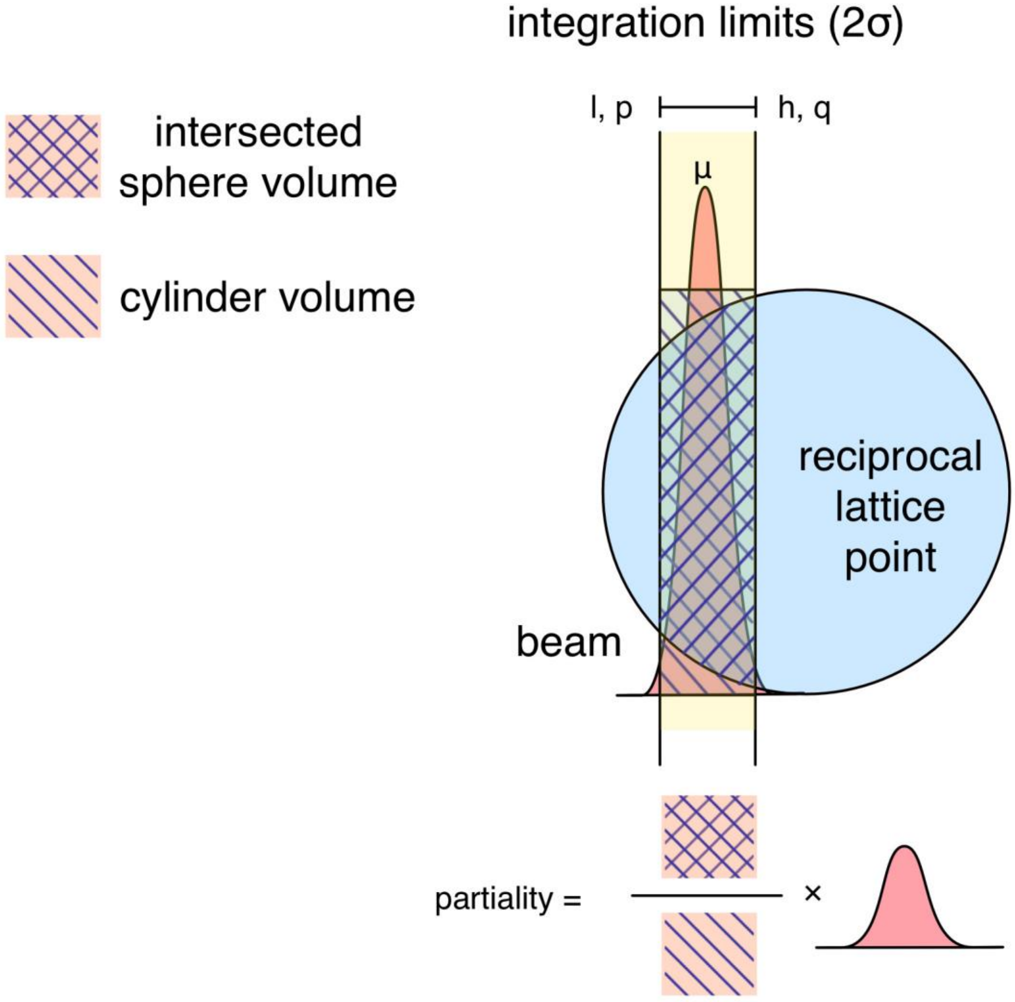


**Supplementary figure 5. Diagram showing the interaction of the Gaussian wavelength distribution with a finite rlp.** Terms are defined in the main text, Methods.

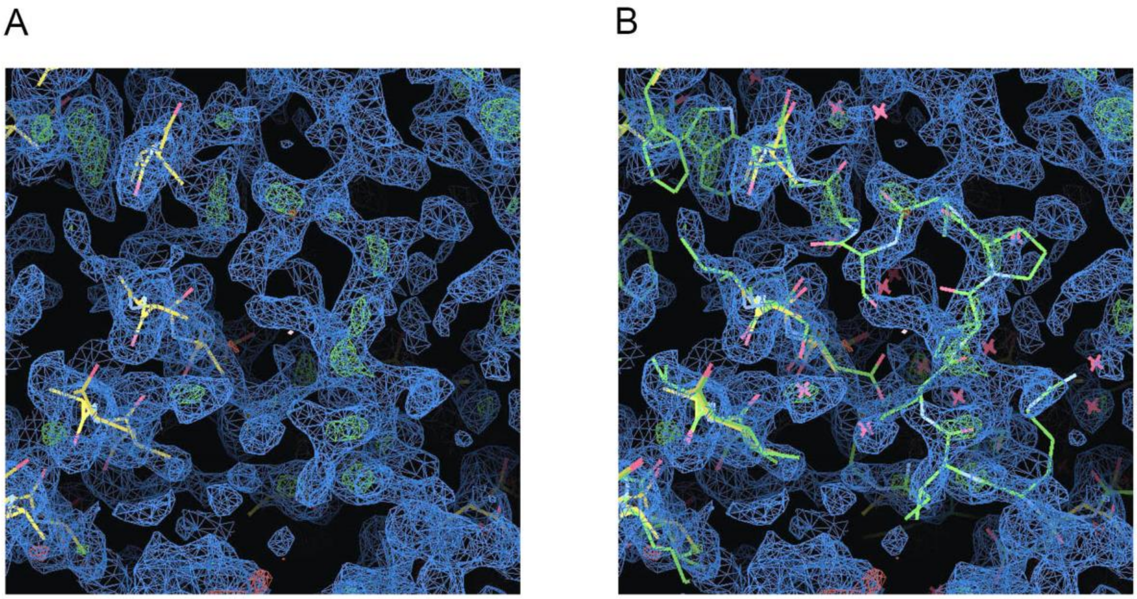


**Supplementary figure 6. Initial molecular replacement electron density map.** (**a**), polyalanine initial model (**b**), in addition the final refined structure is shown.

**a**

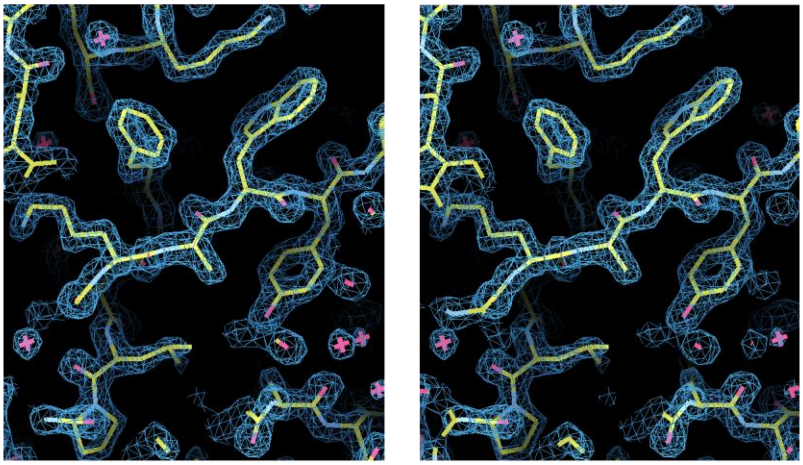

**b**

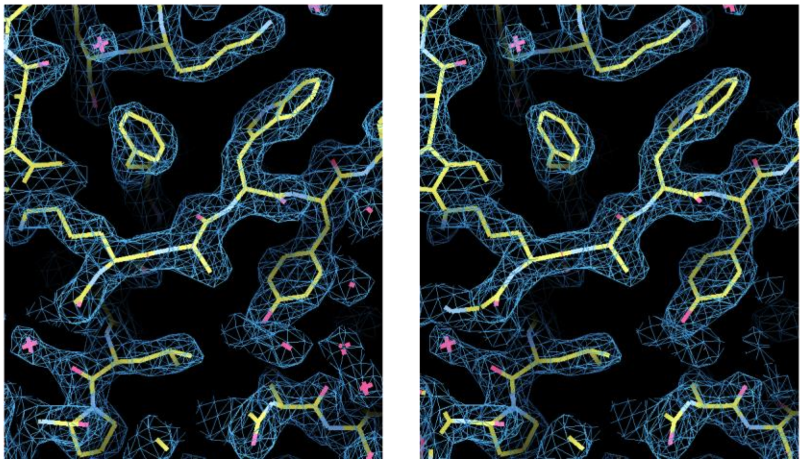

**Supplementary figure 7. Stereo images.** (**a**), XFEL and (**b**), synchrotron structures. As a point of reference the tryptophan in the centre is residue 163. The contour levels for (**a**) and (**b**) are 1.2 σ.

## Supplementary References


1. Stuart, D. I., Levine, M., Muirhead, H. & Stammers, D. K. Crystal structure of cat muscle pyruvate kinase at a resolution of 2.6 A. *J. Mol. Biol.* **134**, 109-142 (1979).

2. Gouet, P., Courcelle, E., Stuart, D. I. & Metoz, F. ESPript: analysis of multiple sequence alignments in PostScript. *Bioinformatics* **15**, 305-308 (1999).
